# Modifications of the Protein Characteristics of Pacaya Caused by Thermal Treatment: A Spectroscopic, Electrophoretic and Morphological Study

**DOI:** 10.3390/polym12051016

**Published:** 2020-04-30

**Authors:** Jocelyn Blanca Esthela Hernández-Castillo, Aurea Bernardino-Nicanor, María de los Ángeles Vivar-Vera, José Luis Montañez-Soto, Gerardo Teniente-Martínez, José Mayolo Simitrio Juárez-Goiz, Leopoldo González-Cruz

**Affiliations:** 1 Doctorado en Ciencias en Ingeniería Bioquímica, Tecnológico Nacional de México/IT de Celaya, Antonio García Cubas Pte. #600 esq. Av. Tecnológico, Celaya 38010, Guanajuato, Mexico; ibq_jocelynn@hotmail.com; 2 Tecnológico Nacional de México/IT de Celaya, Antonio García Cubas Pte. #600 esq. Av. Tecnológico, Celaya 38010, Guanajuato, Mexico; aurea.bernardino@itcelaya.edu.mx (A.B.-N.); gera_tm@hotmail.com (G.T.-M.); jmayolo@gmail.com (J.M.S.J.-G.); 3 Tecnológico Nacional de México/IT de Tuxtepec, Av. Dr. Víctor Bravo Ahuja S/N Col. 5 de Mayo, Tuxtepec 68350, Oaxaca, Mexico; angelesvivar@hotmail.com; 4 Centro Interdisciplinario de Investigación para el Desarrollo Integral Regional del Instituto Politécnico Nacional, Unidad Michoacán, Justo Sierra N°28, Jiquilpan 59510, Michoacán, Mexico; montasoto@yahoo.com.mx

**Keywords:** pacaya, thermal treatment, protein modification

## Abstract

The inflorescences of *Chamaedorea tepejilote* Liebm. are consumed as food in Central America and southern Mexico but is an underutilized food because of its sensory characteristics, principally due to its bitter taste. However, the inflorescences of *Chamaedorea tepejilote* Liebm. are nutritionally promising due to their high protein content (approximately 25%). Protein isolates from pacaya were modified via three different thermal treatments to determine the effect of the treatments on the protein structures. Scanning electron microscopy indicated that the pacaya protein isolate particles had less rough and irregular surfaces with larger particle sizes due to an aggregation process when a thermal treatment was used compared to those when no thermal treatment was used. An increase in the intensity of the low molecular weight protein fractions (≤20 kDa) in the electrophoretic pattern of the proteins was observed, which was generated by the hydrolysis of the proteins by heat treatment. The modifications in the FT-IR spectra showed that thermal treatment of pacaya affected the secondary structure of its proteins, mainly when microwave treatment was used. Raman spectroscopy revealed that the α–helical structure was dominant in the proteins of pacaya and that thermal treatment increased the fraction of the β–sheet structure at the expense of the α–helical structure.

## 1. Introduction

*Chamaedorea tepejilote* Liebm. is an important source as a traditional food in Central America and southern Mexico [1]; however, this plant has a bitter taste and is only consumed by inhabitants that enjoy bitter tastes [2]. On the other hand, *Chamaedorea tepejilote* Liebm is also used in Mexican traditional medicine for the treatment of respiratory diseases, such as coughs, bronchitis, pneumonia and colds [3]. It has also been reported that *Chamaedorea tepejilote* Liebm, at doses of 300 mg/kg, has a similar hypoglycemic activity to that of tolbutamide in in vivo tests [4]. For the treatment of respiratory ailments, two active compounds were isolated from *Chamaedorea tepejilote* Liebm (ursolic acid and oleanolic acid) [5].

However, despite the importance of *Chamaedorea tepejilote* Liebm as a food source, very little has been published on the subject; several studies have focused only on the protein content of powdered, dried inflorescences of *Chamaedorea tepejilote* Liebm, which ranges between 24.19 and 26.72 %; for this reason, pacaya have been considered an important source of protein [1,6,7]. The inflorescences of *Chamaedorea tepejilote* Liebm are generally consumed once they are cooked, which softens the inflorescence tissue, reduces the bitter taste and reduces enzymatic activity, but thermal treatment could induce modification of the protein structure since it has been reported that heating causes modifications in the structure/conformation of proteins, depending on the thermal processing, the number of complementary treatments and the operating conditions applied [8]. Some authors have indicated that high intensity ultrasound (HIU) also modified the physicochemical properties of protein isolates [9], and some studies have reported that changes in the molecular structure of proteins were observed after HIU treatment, principally in the secondary structure, free sulfhydryl groups and particle size [10].

On the other hand, thermal treatment induces several reactions causing modification of proteins, mainly during hydrolysis reactions that generate depolymerization, followed by grouping, which involves the formation of new bonds between protein fractions; large aggregates form by coagulation and are stabilized by disulfide bonds [11]. Furthermore, thermal denaturation of proteins generates several changes in their secondary structures, modifying the physicochemical properties of the proteins, including gelation and water holding capacities [12]. However, few studies have reported on the effect of several thermal treatments on protein structures using Fourier Transform-Infrared Spectroscopy (FT-IR) and Fourier Transform-Raman Spectroscopy (FT-Raman) to determine changes in the proteins after thermal treatment. Thus, because pacaya is commonly consumed in cooked form, this study used both FT-IR and FT-Raman spectroscopy in addition to Scanning Electronic Microscopy (SEM) and Sodium Dodecyl Sulfate–Polyacrylamide Gel Electrophoresis (SDS-PAGE) to determine the effects that the thermal treatment has on the structure of proteins.

## 2. Materials and Methods 

### 2.1. Vegetal Material

*Chamaedorea tepejilote* inflorescences (pacaya) were purchased from a local market in Tapachula, Chiapas, Mexico. The inflorescences were brought to the laboratory in a cardboard box. In the laboratory, the bracts that covered the inflorescence were removed, and only yellow inflorescences were selected. The inflorescences were cut into approximately 0.5 cm cubes, packed in lots of 300 g in PVC bags and stored in an LG Model GR-452SH refrigerator (LG electronics, Monterrey, NL, México) at 4 °C for no more than 24 h.

### 2.2. Thermal Treatments

#### 2.2.1. Hydrothermal Processing

A 300 g sample of pacaya packed under vacuum in a PVC bag was placed in a water bath at 90 °C for 15 min. After the hydrothermal treatment, the sample was cooled at room temperature and then frozen at −20 °C and allowed to set for 24 h until the lyophilization process was conducted [13].

#### 2.2.2. Steaming at Elevated Pressure

A 300 g sample of pacaya packed under vacuum in a PVC bag was placed on a tray in a pressure cooker and steamed for 15 min at 125 °C and under a pressure of 124,106 Pa. After pressure treatment, the sample was cooled to room temperature and then cooled to −20 °C for 24 h prior to being lyophilized [13].

#### 2.2.3. Microwave Cooking

A 300 g sample of pacaya packed under vacuum in a PVC bag was placed in a GoldStar model MS-157XC microwave oven (LG Corp., Seúl, Korea) powered at 1500 W and with an operating frequency of 2450 MHz for 15 min. Then, the sample was cooled at room temperature and frozen at −20 °C for 24 h [13].

### 2.3. Lyophilization Process

All thermally treated samples were lyophilized using a laboratory freeze-dryer (Scientz-10 N, Ningbo, China). During the lyophilization process, the prefreezing temperature was set to −40 °C. When the cold trap temperature reached −55 °C and the system pressure was reduced to 6 Pa, the lyophilization process was initiated. All freeze-dried samples were pulverized and stored in polyethylene bags until ultrasound treatment.

### 2.4. High Intensity Ultrasound (HIU)-Assisted Extraction of Oil from the Pacaya Samples

Pacaya dispersions (7% *w*/*v*) were prepared by adding freeze-dried powder samples into hexane. The ultrasound treatment was carried out in an ultrasonic bath (Model SB-3200 DTN Loyal Key group Shangai Branch Co., Ltd, Ningbo, China) with a working frequency of 40 kHz and a nominal power of 180 W for 20 min.

Samples without ultrasound treatment were used as controls. In brief, pacaya dispersions (7%, *w*/*v*) were prepared by adding freeze-dried, powdered samples into hexane and then gently stirring overnight at ambient temperature.

The pacaya dispersions were filtered using #40 medium Whatman paper, the pacaya powder was recovered and the remaining solvent was evaporated to dryness at ambient temperature. The recovered pacaya powder was stored in PVC bags until analysis.

### 2.5. Preparation of Protein Isolates

Protein isolates from the pacaya powder samples were obtained via isoelectric precipitation as described by Bernardino-Nicanor et al. [14]. The defatted powder was dispersed in distilled water (1:20) and homogenized by magnetic stirring, adjusting the pH to 11.8 with NaOH (0.1 N); then, the solution was centrifuged at 6000 rpm for 30 min at a temperature of 4 °C, and the supernatant was collected. The pH of the collected supernatant was adjusted to pH 4 (HCl; 0.1 N), the precipitated protein was recovered by centrifugation at 3000 rpm for 30 min, and the recovered protein was dried using a forced convection-drying oven (Binder, Model FD115-UL, Bohemia, NY, USA) at a temperature of 50 °C for approximately 6 h.

### 2.6. Characterization of Pacaya Protein Isolates

#### 2.6.1. Proximate Analysis

The proximate analysis of pacaya protein isolates was carried out according to the method of the AOAC (1990) [15]: 925.09 for moisture content; 955.04 for protein content (N × 6.25); 920.39 for lipid content; and 923.03 for ash content. Total carbohydrate content was determined by the difference, from the contents of the other components. Results on dry weight basis were expressed.

#### 2.6.2. Scanning Electron Microscopy (SEM)

Samples of pacaya protein isolates were examined using a JEOL scanning electron microscope (model JEOL, JSM-6300 Akishima, Tokyo, Japan) fitted with a Kevex Si(Li) X ray detector. The analyses were performed under vacuum at an accelerating voltage of 20 kV. The samples were mounted on double-sided carbon tape and covered with approximately 10 nm of gold using a Denton sputter coater [16].

#### 2.6.3. Sodium Dodecyl Sulfate-Polyacrylamide Gel Electrophoresis (SDS-PAGE)

SDS–polyacrylamide gel electrophoresis was performed in 10 g/100 mL separating gels with 4 g/100 mL stacking gels according to the method of Laemmli [17] using the Mini Protean 3Cell (Bio-Rad Laboratories, Hercules, CA, USA) vertical unit. Molecular masses of the polypeptides were calculated using the following standard proteins (Bio-Rad Laboratories Hercules, CA, USA): phosphorylase b (94 kDa), bovine serum albumin (67 kDa), ovalbumin (45 kDa), carbonic anhydrase (30 kDa), trypsin inhibitor (20.1 kDa) and α-lactalbumin (14.4 kDa). The protein samples were dissolved in sample buffer (0.1 mol/L Tris-HCl, pH 6.8, 20 mL/100 mL glycerol, 2 g/100 mL SDS and 0.05 g/100 mL bromophenol blue). Gels were fixed and visualized with either Coomassie Brilliant Blue or silver staining.

#### 2.6.4. Fourier Transform-Infrared Spectroscopy (FT-IR)

The FT-IR spectra of the pacaya protein isolates were acquired on a Perkin Elmer FT-IR spectrophotometer (Perkin Elmer, Inc., MA, USA) using potassium bromide (KBr) discs prepared from powered samples mixed with dry KBr. The spectra were recorded (16 scans) in transmission mode at a resolution of 4000 to 400 cm^−1^ [16].

#### 2.6.5. Fourier Transform-Raman Spectroscopy (FT-Raman)

FT-Raman measurements were performed on a Perkin-Elmer (Perkin Elmer, Inc., MA, USA) 2000R NIR FT-Raman spectrometer equipped with a Nd:YAG laser emitting at a wavelength of 1064 nm and an InGaAs detector. For these analyses, a 180° backscattering refractive geometry was used. The spectrometer was managed using Perkin–Elmer Spectrum software. The spectral data for the pacaya protein isolates were obtained at a wavenumber resolution of 4 cm^−1^ at a nominal laser power of 500 mW. For each spectrum, 20 scans were accumulated to ensure an acceptable signal-to-noise ratio. All Raman spectra were collected at room temperature [16].

## 3. Results and Discussion

### 3.1. Characterization of Pacaya Protein Isolates

The proximate analysis of pacaya protein isolates showed similar content of the fraction (Table 1) on a dry basis, however the protein content in all pacaya protein isolates obtained of flour with different thermal treatments, was low compared to of commercial crops as soy, chickpea, pea, lentil and faba bean isolates [18]. The lipid content for all pacaya protein isolates were low (< 1%) this is concordant with those reported by Leyva-Lopez et al., [19], who indicate that defatting prior to the isolate obtention decreases the lipid content. On the other hand, apparently the isoelectric precipitation technique results in salt formation and for this reason a high-level ash content in the isolates was observed.

### 3.2. Scanning Electron Microscopy (SEM)

**Figure 1:**
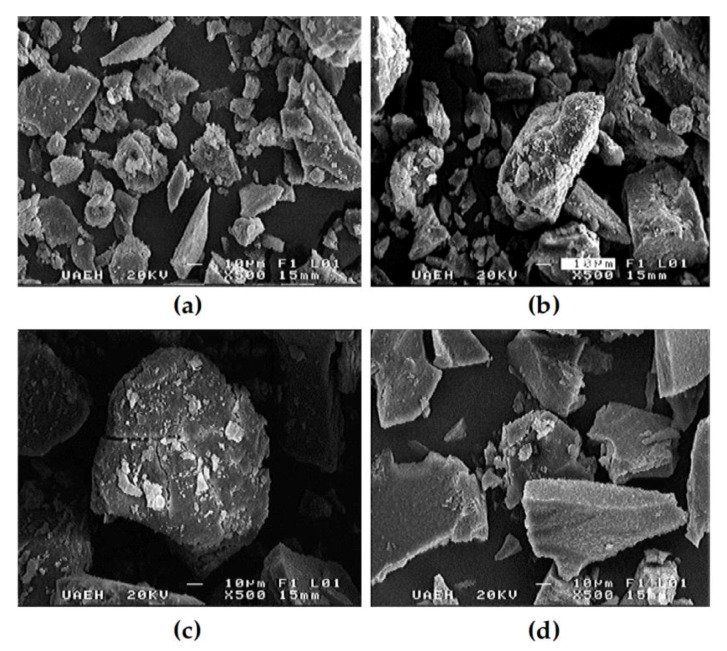
SEM micrograph of pacaya protein isolate (**a**) extracted from pacaya without thermal treatment; (**b**) extracted from pacaya under hydrothermal processing; (**c**) extracted from pacaya via steaming at an elevated temperature; (**d**) extracted from pacaya under microwave treatment.

Figure 1 shows the microstructural characteristics of the pacaya protein isolates with and without thermal treatment. Compared to the pacaya protein isolate without thermal treatment (Figure 1a), thermally treated pacaya protein isolates showed less rough and irregular surfaces (Figure 1b–d). All thermal treatments showed aggregation between the particles of the pacaya protein isolates; for this reason, an increase in the particle size was observed. In the protein isolate obtained from the pacaya thermally treated via microwave, the highest aggregation level was observed. Our findings are in accordance with those of Ketnawa and Liceaga [20], who also found that microwave heating induced this phenomenon in proteins; in addition, Carbonaro et al. [21] reported that microwave cooking increased the association level of proteins in lentil flour. On the other hand, Ma et al. [22] indicated that boiling caused the formation of aggregates of the proteins of pulse seeds. Apparently, any thermal treatment generates a particle aggregation process at different levels, and for this reason, the particle size is modified. The modification in the particle size could be a consequence of the gelation process generated by thermal treatment or by sonication, particularly when sonication is carried out for longer periods of time, leading to the unfolding of proteins, increasing the exposure of hydrophobic groups and free SH groups at the surface of the molecules, which permits interaction with each other to form larger aggregates [23].

The isolate obtained from pacaya flour without thermal treatment showed flaky plate like structures, similar results were reported by Mao and Hua [24] for isolates produced from walnut (*Juglans regia* L.) who reporting that isoelectric precipitation technique change the microstructure of protein. On the other hand, the increase in the size of pressure-treated samples is concordant with those of Ahmed et al., [25], who indicate that high pressure treatment increases the particle size of kidney bean protein isolate. The differences observed in the shape particle of the isolates could be attributed to specific modifications by effect of thermal treatment on the protein fractions constitution. In addition, Chavan et al. [26] mentioned that the protein fractions generate different topographical characteristics as, plate-like or irregular forms and for this reason, the morphological characteristics of the pacaya isolates is the combination of the morphological characteristics of their constitutive fractions.

### 3.3. Sodium Dodecyl Sulfate-Polyacrylamide Gel Electrophoresis (SDS-PAGE) 

**Figure 2:**
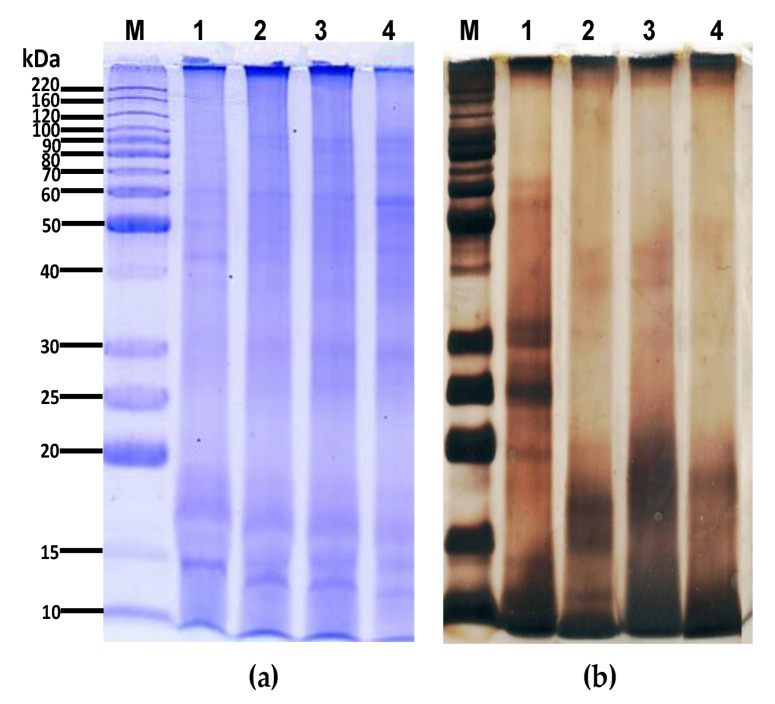
Effect of thermal treatment on the SDS-PAGE pattern of pacaya proteins in (**a**) pacaya flour; (**b**) pacaya protein isolate. Lanes: M: molecular weight marker, 1: extracted from pacaya without thermal treatment, 2: extracted from pacaya under hydrothermal processing, 3: extracted from pacaya via steaming at an elevated temperature, 4: extracted from pacaya under microwave treatment.

The protein components from flours obtained from pacaya with or without thermal treatment were analyzed by electrophoresis (Figure 2a). The heat treatment of pacaya induced protein hydrolysis on at least two subunits (50 kDa and 30 kDa) and generated a dissociation process that led to the formation of several new bands; the dissociation of pacaya protein to yield subunits with greater mobility increased the intensity of bands in the region between 10 kDa and 20 kDa. On the other hand, slight changes in the protein components from the flour obtained from pacaya after steaming at an elevated temperature were observed, which were probably due to the solubility modification of individual proteins [27].

The isolation of proteins was carried out to compare the electrophoretic pattern of the protein isolate (Figure 2b) with those of the total proteins from the defatted flour of pacaya with or without thermal treatment. No similar profiles of protein fragmentation in the electrophoretic protein pattern of the protein isolate and total protein were observed. Modifications in the intensity and distribution of the bands were observed for the thermally treated samples; the isolation process generated a progressive and significant reduction in the number of subunits of high molecular weight. As revealed by SDS-PAGE in the isolation process of the pacaya protein by isoelectric focusing, the basic and acidic conditions used dissociated several bonds, and a considerable amount of protein fractions were detected in the low molecular weight region (between 10 kDa and 20 kDa).

The higher intensity bands that appeared at the lower molecular weight position in the SDS-PAGE of flours obtained from pacaya after thermal treatment indicate the formation of large molecular aggregates and suggest an increase in the low molecular weight protein fractions due to the thermal treatment. Our results are in agreement with the results of Raikos et al., [28], who indicated that when a protein is heat-treated at 80 °C and above, the aggregation process of protein fractions is due to the formation of covalent disulfide bonds via sulfhydryl-disulfide interchange.

In the pacaya protein isolates, the increase in the intensity of the low molecular weight bands was apparently due to the acquisition conditions since during the isolation of the pacaya proteins by isoelectric point, higher solubility was reached at pH 11, while the isoelectric point was reached at pH 2. Some authors indicate that some acidic and alkaline treatments generate the dissociation of proteins and generate new low molecular weight bands [29]. On the other hand, the unfolding of proteins exposes their hydrophobic and sulfhydryl groups, which could interact with each other, allowing for aggregation [30]. Second, both intramolecular hydrogen bonds and non-polar bonds are cleaved and can be reformed during thermal treatment [31], generating an aggregation process.

The electrophoretic pattern obtained in this study, was similar to obtained by other authors, that mentioned that the electrophoretic pattern of palm proteins could has several bands originated by the extraction method, variation between seed storage proteins, varieties, part of the plant analyzed, polymorphism and cultivation origin [32]; however, in all palm samples tested, a high number of bands were observed. For this reason, they were grouped according their distribution pattern, the first group, bands between 0 to 20 kDa; the second group, bands between 20 and 50 kDa; third group, bands between 50 and 120 kDa and the fourth group, bands higher to 120 kDa [33]. However, the thermal treatment induced the disaggregation and unfolding of the proteins resulting in much smaller aggregates generating an increase in the intensity of bands of low molecular weight.

### 3.4. Fourier Transform-Infrared Spectroscopy (FT-IR) Analysis of Structural Changes Due to Thermal Treatment

**Figure 3:**
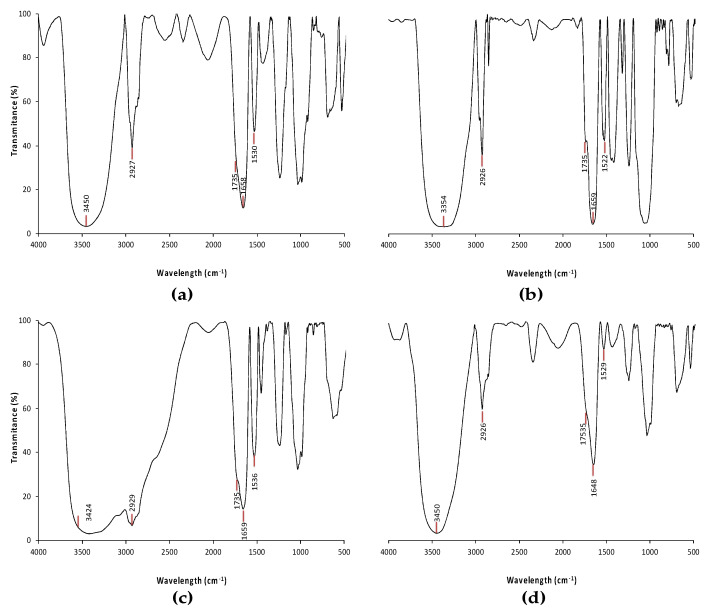
FT-IR patterns of pacaya protein isolates (**a**) extracted from pacaya without thermal treatment; (**b**) extracted from pacaya under hydrothermal processing; (**c**) extracted from pacaya via steaming at an elevated temperature; (**d**) extracted from pacaya under microwave treatment.

The FT-IR spectra for the protein isolate from thermally treated pacaya are shown in Figure 3. Information on molecular vibrations was obtained from similar frequency regions. For this reason, secondary structural changes in the FT-IR spectra were estimated by the modifications in the amide I region (1600–1700 cm^−1^) and the amide II region (1500–1600 cm^−1^).

The amide I region (1600–1700 cm^−1^) shows a defined band around 1655 cm^−1^ that is assigned to the C=O stretching/hydrogen bonding coupled with COO and corresponds to the α-helix structure [34,35]. For the band at approximately 1655 cm^−1^ that appeared after hydrothermal processing, there were no significant differences in comparison with the same band observed for the sample without thermal treatment. However, the protein isolate obtained from the pacaya treated with microwave showed a decrease in the 1655 cm^−1^ band intensity (Figure 3c,d), indicating a loss of the α-helix protein structure.

In the amide II region (1480–1575 cm^−1^), a prominent band at approximately 1530 cm^−1^ was observed in most pacaya protein isolates. According to some authors, this band corresponds to the bending vibrations of N–H groups and the stretching vibration of C–N [36,37].

The thermal treatments enhanced the small shoulder near the 1735 cm^−1^ region, which indicates that small amounts of lipids were present in the protein isolate of the pacaya [36,38].

On the other hand, a relatively flat peak near 3400 cm^−1^ was observed in the protein isolate obtained from pacaya treated with steam and pressure, suggesting that more hydrogen bonds formed in the sample, generating a band near 2930 cm^−1^, which was attributed to the C–H stretching vibration of –CH_2_– from the amino acid interacting with the pacaya starch molecules at the C–1 position of the reducing end during a retrogradation process in the starch [39,40].

In the spectra of the protein isolate obtained from the pacaya treated with microwave, a decrease in the intensity of the amide II band was observed, apparently due to aggregation of the pacaya protein isolate caused by the thermal treatment, which generated a gelation process. According to other authors, protein isolates are most sensitive to physicochemical treatments than complete food samples, reporting a decrease or disappearance of the amide II band [36]. It has been reported that microwave heating on soy isolates, first induces disaggregation and unfolding of proteins and later can form much smaller aggregates that permit an increase in the collision between protein and saccharide molecules [41], and apparently this effect increases this signal in the FT-IR spectra 

Several authors have reported that the disappearance of the band near 1735 cm^−1^ is caused by the removal of lipids [36,38], several authors indicate that defatting prior to isoelectric precipitation technique leads to improved protein extraction and lipid reduction as protein—lipid interactions are significantly reduced, similar results by Karaca et al. [18] and Leyva-Lopez et al. [19] in legumes and cereals, respectively were observed.

The modification observed in the protein isolate obtained from pacaya treated with steam and pressure to the 3400 cm^−1^ band was likely due to the conjugation of the protein with the starch induced by the thermal treatment [39,40]. Similar results were obtained with a restricted amount of water and high temperatures that promoted the protein damage and rearrangement of the structures that led a shrinkage of their tertiary structure [42] generating a higher exposition of other remain components mainly the amylose an amylopectin molecules.

### 3.5. Fourier Transform-Raman Spectroscopy (FT-Raman) Analysis of Structural Changes Due to Thermal Treatment

**Figure 4:**
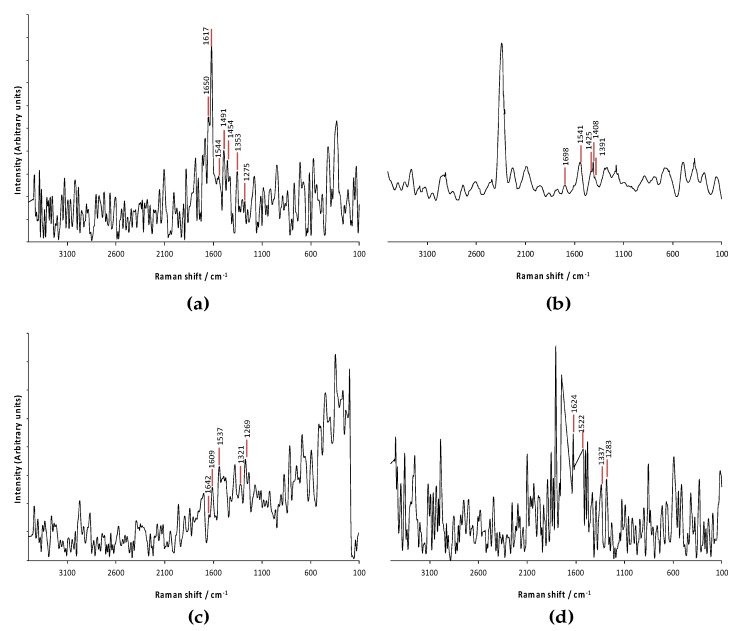
FT-Raman spectra of pacaya protein isolates (**a**) extracted from pacaya without thermal treatment; (**b**) extracted from pacaya under hydrothermal processing; (**c**) extracted from pacaya via steaming at an elevated temperature; (**d**) extracted from pacaya under microwave treatment.

The Raman spectra of the uncooked and thermally treated samples (hydrothermal processing, steam pressure and microwave) are presented in Figure 4. Minor variations in the Raman spectra were observed in the protein isolate obtained from the pacaya treated thermally with microwave (Figure 4d). The Raman spectra of the pacaya protein isolate obtained from the uncooked pacaya showed C=O stretching vibrations and N–H wagging in the peptide bonds coming from the amide I group at approximately 1650 cm^−1^ [43]. Comparison of the amide I band of the protein isolates reveals that the protein isolate obtained from uncooked pacaya possessed a predominant α–helical structure (1650 cm^−1^) with a minor contribution of the β–sheet structure (1635 cm^−1^). 

The Amide II region in the protein isolate obtained from the pacaya without thermal treatment was observed at approximately 1540 cm^−1^ and corresponded to N–H bending [36]. An enhancement in the amide II band in the protein isolate obtained from the pacaya with hydrothermal processing was observed, while in the protein isolates obtained from the pacaya treated with steam at an elevated pressure and with microwave cooking, slight modifications in the amide II band were observed.

On the other hand, the amide III region in the pacaya protein isolates was observed at approximately 1280 cm^−1^ and corresponded to C–N stretching, N–H bending and C–C stretching [44].

After thermal treatments, the intensities of the bands attributing to the α–helical structure (around 1650 and 1340 cm^−1^) decreased. These patterns were consistent with other studies, and a transitional effect from the α–helical structure to the β–sheet structure occurs during protein gelation [36,45].

For the protein isolate obtained from uncooked pacaya, the α–helical structure was dominant. Thermal treatment generated modifications in the amide III region that indicate that the secondary structure changed from an α–helical structure dominant form to a more complex structure with an increase of the β–sheet structure and disordered structures. These results confirm the modifications observed in the amide I region in this study and are in accordance with previously published reports that indicated that thermal treatment samples improved the fraction of the β–sheet structure at the expense of the α–helical structure [46].

## 4. Conclusions

The results obtained in this study are important as thermal treatments may induce important structural changes which could have significant impacts on the physicochemical properties of the isolates; for this reason, the main findings obtained could be used in future studies to correlate structural modifications and functional properties of the pacaya protein isolates.

Thermal treatment used as a cooking method results in grouping of protein particles, modifying the observed particle size and generating a less rough granular structure. The electrophoretic pattern showed that the polypeptidic composition of pacaya protein isolates was modified by thermal treatment, generating a higher concentration of the low molecular weight fractions. Modifications in the FT-IR and FT-Raman spectra were observed due to the effect of the thermal treatment on the proteins. The two applied spectroscopic methods were complementary and allowed for the identification of modifications in the secondary structure of pacaya proteins.

## Figures and Tables

**Table 1:** Chemical composition of the pacaya protein isolates obtained of flour with different thermal treatments.

Previous Thermal Treatment	Protein (%)	Moisture (%)	Lipid (%)	Ash (%)	* Carbohydrates (%)
Without thermal treatment	75.27 ± 2.03 ^d^	4.00 ± 0.33 ^d^	0.59 ± 0.07 ^a^	5.67 ± 0.34 ^a^	14.55 ^c^
Hydrothermal processing	76.46 ± 3.45 ^a^	4.15 ± 0.41 ^b^	0.56 ± 0.08 ^c^	4.25 ± 0.41 ^d^	14.80 ^b^
Steaming at elevated pressure	75.51 ± 3.85 ^c^	4.09 ± 0.15 ^c^	0.55 ± 0.08 ^d^	4.75 ± 0.75 ^c^	15.25 ^a^
Microwave process	75.96 ± 5.25 ^b^	4.23 ± 0.27 ^a^	0.58 ± 0.09 ^b^	5.50 ± 0.33 ^b^	13.90 ^d^

Different letters in each column indicate significant differences at *p* < 0.05. * Determined by difference.
